# Nuclear processes associated with plant immunity and pathogen susceptibility

**DOI:** 10.1093/bfgp/elv013

**Published:** 2015-04-06

**Authors:** Graham B. Motion, Tiago M.M.M. Amaro, Natalja Kulagina, Edgar Huitema

**Keywords:** nucleus, immunity, pathogen, effector, susceptibility, next-generation sequencing (NGS)

## Abstract

Plants are sessile organisms that have evolved exquisite and sophisticated mechanisms to adapt to their biotic and abiotic environment. Plants deploy receptors and vast signalling networks to detect, transmit and respond to a given biotic threat by inducing properly dosed defence responses. Genetic analyses and, more recently, next-generation -omics approaches have allowed unprecedented insights into the mechanisms that drive immunity. Similarly, functional genomics and the emergence of pathogen genomes have allowed reciprocal studies on the mechanisms governing pathogen virulence and host susceptibility, collectively allowing more comprehensive views on the processes that govern disease and resistance. Among others, the identification of secreted pathogen molecules (effectors) that modify immunity-associated processes has changed the plant–microbe interactions conceptual landscape. Effectors are now considered both important factors facilitating disease and novel probes, suited to study immunity in plants. In this review, we will describe the various mechanisms and processes that take place in the nucleus and help regulate immune responses in plants. Based on the premise that any process required for immunity could be targeted by pathogen effectors, we highlight and describe a number of functional assays that should help determine effector functions and their impact on immune-related processes. The identification of new effector functions that modify nuclear processes will help dissect nuclear signalling further and assist us in our bid to bolster immunity in crop plants.

## Introduction

Plants are continuously challenged by biotic and abiotic stresses. As sessile organisms they have evolved refined mechanisms to deal with those threats that affect plant health or survival. Decades of intense genetic studies on the processes underpinning growth and reproduction, plant development, adaptation to stress as well as the mechanisms of immunity and susceptibility to pathogens has led to significant insights and critically unveiled common mechanistic themes in seemingly distinct processes [1]. The emergence of near complete genome sequences, next-generation sequencing (NGS) technologies and advanced functional assays in plants, has pushed forward those boundaries by allowing systems level investigations of traits and processes that were unveiled by genetic and biochemical studies. This is particularly true in the plant–microbe interactions field, where the availability of genome sequences for an increasing number of hosts and pathogens together with a facile and powerful set of functional assays has led to a greater understanding of immunity and pathogen susceptibility in plants. These advances, in turn, have led to the emergence of conceptual models that explain immunity, susceptibility and host–microbe co-evolution [2–4].

Within the natural environment, plants are constantly challenged by a diverse array of microbes but infection only occurs sporadically. As a mechanism to avoid infection, plants deploy membrane-bound pattern recognition receptors (PRRs) that bind and recognize microbe-derived molecules [Microbe or Pathogen Associated Molecular Patterns (M/PAMPs)] or host-derived damage-associated molecules [Damage Associated Molecular Patterns (DAMPs)]. In this scenario, recognition of microbial patterns or the detection of host-derived damage signals leads to the rapid induction of signalling cascades that ultimately lead to the induction of defence gene expression and activation of plant defence responses. Collectively, these processes lead to enhanced immunity to most microbes designated as PAMP-triggered immunity (PTI) [2, 5]. Consistent with a prominent role for pattern perception in plants, an increasing number of M/PAMPs have been identified in a wide range of divergent organisms. These include peptidoglycans (PGNs) and Lipopolysaccharides (LPS) from bacterial cell envelopes, bacterial elongation factor thermo unstable (EF-TU [6]), flg22 (a 22 amino acid peptide derived from bacterial flagellin), chitin from fungal cells walls and glucans as well as glycoproteins in oomycetes [7]. The observation that many microbial molecules are recognized suggests that pattern recognition is one key feature that shapes induced immune responses in plants.

By definition, microbial pathogenesis must feature compromised structural barriers and the prevention or suppression of induced immune responses of the infected host. The vast majority of plant pathogenic microbes achieve invasion and colonization by deploying secreted protein repertoires (effectors) that help degrade cellular structures, limit perception and suppress defence signalling. Collectively, these effectors reprogram cells into an immunocompromised state referred to as effector triggered susceptibility (ETS) [2]. Given the importance of pathogen-encoded virulence factors in disease, genome sequencing and functional genomics studies have allowed the rapid identification of pathogen effector repertoires, many of which have been subject to intense study. Critically, these effectors either function in the apoplast (apoplastic or extracellular effectors) or traffic into the host cell (cytoplasmic or intracellular effectors) [8] where they perturb or modify cellular processes required for immunity. Intracellular effectors have been identified in a wide range of pathogenic microbes among which many appear to target the nucleus or proteins destined to function in this compartment [9]. Taken together, these and other observations have led to the suggestion that the host nucleus is an important compartment where the fate of plant–microbe interactions is determined [9, 10].

**Figure 1:**
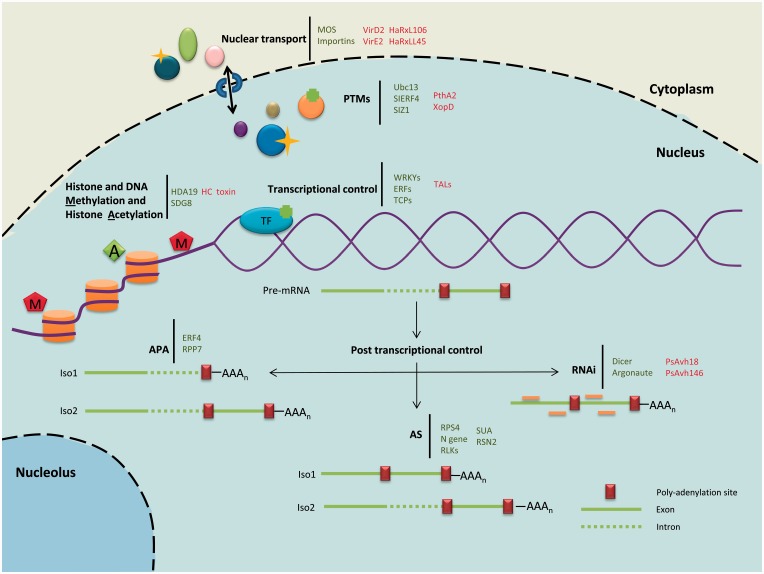
Schematic representation of immunity-related nuclear processes. Several nuclear processes have been implicated in plant immunity. Plant proteins involved in plant immunity are highlighted in green and pathogen effectors that target host nuclear processes are highlighted in red. Nuclear transport: The transport of immune regulators and signalling proteins into the nucleus is the first step of the nuclear involvement in immunity. Nuclear transporters as importins and proteins of the MOS family have been involved in plant resistance mechanisms. Moreover, these transporters were shown to be required for the transport of nuclear effectors. PTMs: PTMs are also believed to be an essential mechanism to regulate defence responses in the cytoplasm but also in the host nucleus. Phosphorylation, ubiquitination and sumoylation have been shown to target nuclear proteins as WRKY transcription factors and thus regulate defence responses. Histone and DNA methylation and histone acetylation: Chromatin remodelling modifications like histone acetylation (A) and histone and DNA methylation (M) have also been recently connected with immunity. These modifications are thought to alter chromatin structure and therefore alter gene expression during a defence response. Transcriptional control: During an infection process, dramatic transcriptional changes occur. These changes mediated in part by transcription factors (TFs) are crucial for a proper plant immune response. Post-transcriptional control: Immunity appears to be regulated not only at the post-translational and transcriptional level but also at the post-transcriptional level. Processes as APA, AS and RNAi have also been connected with plant immunity. (A colour version of this figure is available online at: http://bfg.oxfordjournals.org)

In this review, we will summarize and highlight exciting advances in our understanding of nuclear processes that underpin plant immunity (Figure 1). We will also describe the means by which some pathogens can modify these processes in their bid to colonize plants. Based on the basic assumption that a process required for immunity can be targeted by a pathogen, we will propose and highlight possible assays that may help define effector functionality in plants.

## Nuclear processes that drive immunity and may be targeted by pathogens

### Nuclear transport

Translocation of immune regulatory and signalling proteins into the nucleus marks the onset of the host nucleus’ involvement in the immunity process. Nuclear trafficking is mediated by importins, exportins and nucleoporins, all of which are involved in transport of cargo across the nuclear envelope [9]. Proteins representing diverse functions are transported into the nucleus and include classes of transcriptional regulators, which form a complex network and direct plant immune responses through transcriptional reprogramming [11]. Interestingly, an increasing number of Resistance (R) proteins such as Resistance to Ralstonia solanacearum 1 (RRS1), N, Mildew Locus A (MLA), resistance to Pseudomonas syringae 4 (RPS4), RX require nuclear transport for activation (reviewed in [12]). Tomato RPS4 recognizes *Pseudomonas syringae* AvrRps4 resulting in nuclear accumulation of the plant immune regulator EDS1 [13], while barley MLA was shown to be directly associated with several transcription factors (TFs), essential for plant defences (WRKY, MYB6) [14]. These findings indicate the crucial role of nuclear trafficking in plant immune signalling.

Given the importance of plant nuclear dynamics for plant immunity, it is not surprising that its components are targeted, imitated or required by pathogens to promote infection. In *Nicotiana benthamiana*, *Xanthomonas campestris* pv. *vesicatoria* AvrBs3 effector was shown to contain a Nuclear Localisation Signal (NLS) and mimic eukaryotic TFs, affecting host cell development [15]. Effector activity can also modify the subcellular localization of their corresponding target proteins. The *Phytophthora infestans* effector Pi03192 (PITG_03192) targets two NAC [NAM (no apical meristem), ATAF, CUC (cup-shaped cotyledon)] TFs, thereby preventing NAC relocalization to the nucleus during infection and after PTI stimulation [16]. Furthermore, *Arabidopsis thaliana* nucleo-trafficking proteins of the Modifier of SNC1 (MOS) family were reported to be involved in plant basal and constitutive resistance [17]. For instance, *Agrobacterium tumefasciens* NLS-containing effectors VirD2 and VirE2 can interact with several importins to translocate bacterial T-DNA into the host nucleus [18, 19]. In the meantime, silencing of Importin α 1 or 2 in *N. benthamiana* was shown to negatively affect the nuclear import of several *P. infestans* effectors [20], suggesting requirement of host machinery by the pathogen. These findings illustrate the critical role nucleo-cytoplasmic transport plays in immunity and how pathogens either co-opt or modify these processes to their benefit.

### Post-translational modifications

Induction of PTI features the quick initiation of signalling cascades at the cell membrane, followed by spatial and temporal channelling of the PTI signal throughout the cell. A key step in this process involves the diffusion or transport of many of these proteins into the nucleus. Given that post-translational modifications (PTMs) are rapid modifiers of the cellular protein complement, protein modification is thought to underpin PTI signal transmission and regulation in the plant cell [21, 22]. With >300 different types of PTMs described thus far, the collective activity of the principal enzymes driving PTMs generate enormous proteome plasticity [21, 23]. Despite their importance, there are few examples implicating PTMs in plant immunity with phosphorylation, ubiquitination and sumoylation having received most attention in recent years.

Phosphorylation of receptor like kinases is crucial for PAMP perception and activation of subsequent mitogen activated protein kinase (MAPK) signalling cascades [21, 24]. In the nucleus, phosphorylation is equally important as TFs required for immunity (e.g. WRKY and ethylene-responsive type TFs) often require phosphorylation for their activation [25]. For example, PTI-mediated activation of MKK4 and MKK5 in *Arabidopsis* leads to resistance against *P. syringae* [26], owing to activation of WRKY-mediated gene expression.

Several effectors were shown to target phosphorylation-mediated signalling events in the host cytoplasm [e.g. AvrPto and AvrPtoB targeting FLAGELLIN SENSING 2 and BRI1-ASSOCIATED KINASE 1; and AvrAC targeting BIK1 and RIPK] [27, 28]. This further demonstrates the importance of kinase-mediated signalling cascades to plant immunity, which can ultimately lead to phosphorylation events in the nucleus to drive changes in host gene expression. If true, nuclear effectors that target phosphorylation events would be highly useful for successful pathogens. CRN8, a nuclear effector from *P. infestans*, was shown to be a functional kinase, and its over-expression in *N. benthamiana* leaves increased *P. infestans* virulence [29]. The molecular target(s) of this and other nuclear effectors, however, are yet elusive, hampering our progress towards understanding the modes of action of these proteins towards immunity.

Ubiquitination involves the reversible conjugation of ubiquitin to specific lysine residues in a target protein. Protein ubiquitination affects many processes and has been firmly connected with immunity-associated signalling events in plants [30, 31]. In line with the importance of ubiquitination as an immunity-associated PTM, a vast range of plant pathogens target this process with their effectors [32]. For example, PthA2, a nuclear type III effector protein from *Xanthomonas axonopodis* was shown to target the host ubiquitin machinery. It interacts with Ubc13, a ubiquitin conjugating enzyme, avoiding K-63 linked ubiquitination required for DNA repair [33]. Interestingly DNA damage was recently proposed to be a conserved mechanism deployed by plant pathogens [34].

Sumoylation is another PTM highly connected with nuclear plant defence mechanisms. This is evident based on the effects of the mutants of a nuclear *A. thaliana* E3 sumo ligase *Siz1*. *siz1* mutants exhibit constitutive expression of pathogenesis-related (PR) and disease response genes leading to increased resistance to the bacterial pathogen *P. syringae* pv. tomato (Pst) DC3000 [35]. XopD, a type III secretion effector from the bacterial pathogen *Xanthomonas euvesicatoria* was shown to target host nuclear sumoylation status. XopD was shown to catalyse the sumo hydrolysis of SIERF4, a tomato ethylene responsive transcription factor, causing its destabilization. The absence of SIERF4 avoids the transcription of ethylene (ET) defence genes required for immunity against *Xanthomonas* infection [36]. It is without question that pathogens target plastic host nuclear proteomes (and their PTM status) to suppress immune signalling. The future challenge will be to identify and further characterize the PTMs mechanisms generally involved in immunity while implicating those regulators and their modification in immunity.

### Chromatin remodelling and DNA modification

Besides inheritance of genes, the modification of histones, chromatin remodelling and DNA methylation provide another means by which plants can pass on beneficial (immunity related) traits to their progeny. N-terminal histone tails are post-translationally modified, e.g. by acetylation, methylation, phosphorylation, ubiquitination, by modifying enzymes recruited by transcription factors. These enzymes create a ‘histone code’, which causes specific changes in chromatin configuration and gene expression. Chromatin configuration is controlled by ATP-dependent chromatin remodelling complexes, which allow or prevent access to DNA by transcription factors to regulate essential cellular processes [37]. These chromatin remodelers contain a conserved SUCROSE NONFERMENTING2 catalytic ATPase domain. These use the energy from ATP hydrolysis to move, remove or form nucleosomes on DNA [38]. DNA methylation is controlled by methyltransferases, which add a methyl group to the fifth carbon of cytosines. Plant DNA methylation patterns can be passed on to progeny to permanently affect genome activity through a variety of processes, ultimately regulating plant immunity [39].

The occurrence of histone modifications at defence-related genes leads to transcriptional regulation of immunity during infection. Two of the best characterized histone modifications are acetylation and methylation. Histone H3/H4 acetylation, which is linked to gene activation, is controlled by histone acetyltransfereases and histone deacetylases (HDACs) [40]. The Arabidopsis HDAC, HISTONE DEACETYLASE19 is induced during *P. syringae* infection to help defence. This occurs by repressing the transcription factors WRKY38 and WRKY62, which normally negatively regulate the expression of PR genes [41]. Histone H3 methylation can be an activating or repressive modification depending on which lysine or arginine residues the methyl group is added to and the number of groups added [42]. The *Arabidopsis* methyltransferase SET DOMAIN GROUP8 is known to activate expression of nucleotide binding-leucine rich repeats (NB-LPR) genes, e.g. LAZARUS5 (LAZ5), which has an activating H3K36me3 mark enriched at the LAZ5 locus during infection [43]. Methylation of H3K4 at the nucleosome of WRKY70 stimulates salicylic acid (SA)-dependent defence responses, which leads to the expression of NB-LRR genes as well as several defence-related TFs [44].

If plant defences are under some degree of epigenetic control, it is reasonable to assume that these processes would be targeted by pathogens. *Cochliobolus carbonum* HC toxin produced during maize infection inhibits HDAC activity, causing hyperacetylation in susceptible, but not resistant, maize plants [45]. It is therefore not surprising that DNA methylation patterns can be altered as a result of pathogen infection. Indeed, *Arabidopsis* tissue infected with *P. syringae* shows massive hypomethylation as a direct result of active demethylation [40]. Although it is now established that pathogen infection alters histone modification, the pathogen-encoded factors responsible for these processes are yet to be identified.

### Transcriptional and post-transcriptional regulation

Transcriptional reprogramming forms a central component of the plant immune response on perception of a pathogen, whether it occurs during PTI or Effector Triggered Immunity (ETI). As part of a robust defence response against pathogen ingress, dramatic transcriptional changes occur, affecting up to 20% of the host gene complement [46]. Transcriptional reprogramming occurs as a consequence of cross-talk between signal transduction pathways that include pattern-induced MAPK signalling cascades, the production and perception of increased levels of SA, jasmonic acid (JA) and interplay with other plant phytohormones [47]. Given the importance of transcriptional reprogramming, targeting gene expression must represent an important strategy that most pathogens use to overcome host immune responses. Indeed, *Hyaloperonospora* *arabidopsidis* has been shown to interfere with plant immunity through the secretion of the nuclear localized effector HaRxL44. HaRxL44 decreases SA immune processes by interfering with mediator function causing a shift towards JA/ET signalling, bringing about enhanced susceptibility to the pathogen [48]. The *Arabidopsis* transcription factor TCP14 (TEOSINTE BRANCHED1, CYCLOIDEA, and PCF), which has roles in plant development as well as regulation of defence genes [49, 50], has been identified as a key target for divergent plant pathogens [51, 52]. This finding further enhances the notion that targeting transcriptional processes is a key strategy used by many pathogens to evade the host immune response. Besides the targeting of host transcription factors, some pathogens secrete effectors that act as transcriptional regulators. *Xanthomonas* species have been shown to use a novel class of effectors, known as transcription activator-like effectors, which function to bind specific host DNA sequences, resulting in the expression of host genes that aid pathogen colonization and infection [53, 54].

### RNA interference

Similar to many other eukaryotes, plants use a sophisticated RNA interference (RNAi) machinery to control the expression of its gene repertoire and to combat viral infection by degrading viral RNA. Generally, plants use RNAi as a defence response in two ways: transcriptional gene silencing (TGS) and post-transcriptional gene silencing (PTGS). During TGS, RNA is recognized by Dicer to produce small interfering RNAs, which are loaded into Argonaute to mediate defence through RNA silencing. PTGS on the other hand involves siRNAs being incorporated into the ARGONAUTE4 containing RNA-induced transcriptional silencing complex, which guides heterochromatin formation and methylation, acting as a positive regulator of plant defence [37, 55]. Both TGS and PTGS are emerging as important regulators of PTI and ETI signalling as well as mediators of R gene silencing. Perhaps not surprisingly, it is now known that besides viruses, bacteria and oomycetes produce effectors that suppress RNA silencing. Qiao *et al*. [56] showed that two *Phytophthora sojae* effectors (PsAvh18 and PsAvh146) inhibit biogenesis of small RNAs to suppress RNA silencing, while Navarro [57] identified effectors from *P. syringae* that can either suppress transcription of PAMP-responsive miRNAs or inhibit miRNA function.

### Alternative splicing

Alternative splicing (AS) is a nuclear process by which a single pre-mRNA is processed into multiple transcript isoforms, providing a source of diversity within eukaryotic transcriptomes and proteomes [58]. AS has also been highly connected with plant defence mechanisms. In a RNA-seq-based study, it was suggested that >90% of expressed genes can be alternatively spliced during *P. syringae* infection in *Arabidopsis* [59], although its functional relevance remains largely unclear. Importantly, AS has been implicated in plant immune responses more directly through the identification of R-gene splice variants for a great number of resistant proteins [60]. Although most of these splice variants have unknown functions, AS allows generation of functionally diverse NB-LRR proteins, providing a raft of conceptual links to immunity that are likely to be explored. Moreover, AS has been connected to nonsense-mediated mRNA decay [61], which is also involved in plant immunity [62, 63].

RPS4, an R protein from *Arabidopsis*, undergoes AS, and the truncated transcripts are required for its immune functions. Removal of RPS4 introns abolished RPS4 function, but transgenic *Arabidopsis* lines expressing intron-deficient and truncated transgenes of RPS4 showed partial resistance to *P. syringae*. This shows that the alternatively spliced variant of RPS4 has immune-related functions [64]. AS of R genes was also shown to be affected by pathogen challenge. The N gene, encoding for an R protein from tobacco, encodes two transcripts via AS: the full length protein (N_S_); and a truncated version lacking 13 of the 14 repeats of its LRR domain (N_L_). The N_S_ transcript is more prevalent before and for 3 h after tobacco mosaic virus (TMV) infection, while the N_L_ version is more prevalent 4–8 h post-infection. As in the case of RPS4, both transcripts of the tobacco N gene were shown to be required for full resistance against TMV infection [65]. Another important class of proteins in the plant immune system that were shown to undergo AS are the plant immune receptors of the receptor-like kinase (RLK) family. In a recent study, two splicing factors, suppressor of abi3-5 (SUA) and required for SNC4-1D 2 (RSN2), were identified as regulators of AS events in two *Arabidopsis* RLKs: suppressor of npr1-1, constitutive 4 (SNC4) and chitin elicitor receptor kinase1 (CERK1). CERK1 is a chitin receptor, and in Arabidopsis mutants lacking SUA and RSN2, the chitin-mediated production of reactive oxygen species is reduced in contrast with the levels of growth of *P. syringae* in these mutants. This suggests that AS of CERK1 is required for its functions in immunity [66].

Despite the importance of AS to plant immunity, no pathogen effectors are known to target this mechanism. Thus, a more in-depth understanding of AS events in plant defence responses and new ways of testing the action of effectors towards AS processes are yet required. Functional assays that report on AS induction or perturbation owing to effector activity could become crucial in understanding plant immunity and susceptibility.

### Alternative polyadenylation

Alternative polyadenylation (APA) is another nuclear-based mechanism used to control gene expression at a post-transcriptional level. Immediately after transcription, pre-mRNAs are capped, spliced and cleaved, allowing the addition of a poly(A) tail to their 3′ ends. This tail is essential for mRNA stability, transport to the cytoplasm and recognition by the translational machinery. The occurrence of APA is dependent on the location at which the pre-mRNA is cleaved [67].

APA appears to be highly present in plants. A deep-sequencing study showed that APA can affect up to 70% transcribed *Arabidopsis* genes [68]. APA has been observed in plants under different developmental stages and when treated with SA. Interestingly, 35% of the genes that undergo APA on SA treatment are related to biotic or abiotic stresses, which may indicate a function for APA in adjusting the expression of certain immunity-related genes [67].

**Figure 2:**
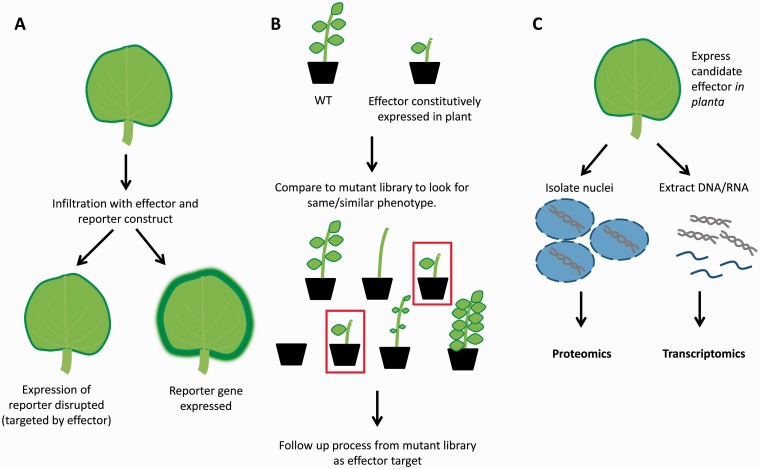
Schematic representation of approaches to identify and characterize nuclear effector functions. (**A**) A construct reporting to the function of an immunity-related process could be over-expressed along with an effector. If the effector targets the tested immunity process, the expression of the reporter would be disrupted. This assay allows the identification of effectors targeting a known host immunity process. (**B**) Constitutive expression of nuclear effectors *in planta* and a phenotypic comparison with previously characterized mutant plants could allow the connection of effectors with specific cellular processes. (**C**) Proteomic and transcriptomic analysis in plants expressing an effector could be useful to further understanding what processes are being targeted by a specific effector. Moreover, it could be a valuable approach to identify new plant nuclear immunity-associated processes. (A colour version of this figure is available online at: http://bfg.oxfordjournals.org)

APA functions in plants are largely unknown with exception to its connections with flowering time control [69]. However, a few recent studies connect APA with plant immunity. A recent study showed that the *Arabidopsis* RNA binding protein FPA, which regulates several APA processes, negatively regulates PTI processes through the immune-related protein ETHYLENE RESPONSE FACTOR 4 (ERF4). ERF4 undergoes APA on flg22 detection in *Arabidopsis* and FPA prevents it [70]. In another study the *Arabidopsis* resistance protein recognition of peronospora parasitica 7 (RPP7) was shown to undergo APA to control its expression in a histone methylation-dependent manner [71]. Largely because the links between APA and immunity have only become clear in recent years, evidence that ties effector action to APA and immunity is lacking. Again, the establishment of assays that allow identification of APA targeting effectors in direct screens (Figure 2) would allow us to probe these processes further.

## Functional genomics in the –omics era: functional assays in plants revisited

It is without question that NGS sequencing and other -omics-driven studies have led to major leaps in our understanding of immunity-associated processes in plants. Given that access to -omics platforms is becoming cheaper, data analyses more straightforward and our understanding of general processes is becoming ever more detailed, we envision that the near future will see proliferation of targeted functional assays designed to identify specific effector functions and test their roles in immunity. We therefore will provide an overview of assays that have recently emerged, are derived from current assays or we deem to be achievable with existing technological platforms. These experimental strategies are summarized in Figure 2.

### Reporter assays

One possible approach to identifying effector functions is the utilization of reporter assays for specific nuclear immunity processes (Figure 2A). A prime example of this approach is the use of a RNA silencing suppression assay [56]. In this assay, RNA silencing is triggered by the generation of a silencing signal against green fluorescent protein (GFP) while co-expressing an effector of interest. In the absence of a silencing suppressor, this would result in little or no GFP fluorescence in the infiltrated leaf area (Figure 2A). However, in the presence of effectors that are capable of suppressing RNA silencing, a strong GFP fluorescence signal is retained when compared against control treatments (no effector). An analogous and other good example is presented in [72], where they used a protoplast assay to identify effectors that target aspects of flg22-induced immunity, including MAP kinase activation and gene expression. This was achieved by looking for the reduction in activity of a flg22-induced luciferase reporter. This allowed the identification of eight effectors, which perturbed flg22-induced immune responses [72].

These examples highlight how specific reporter assays can be used to effectively identify effectors that target a given immune process. Importantly, these types of assays could be adapted to investigate other nuclear immunity processes highlighted in this article. In these scenarios, constructs that report on the occurrence or disruption of AS or APA could easily be implemented. Using this approach, it is possible that this will enhance our understanding of the nuclear immunity processes perturbed by pathogen effectors during the infection process.

### Phenocopy assays

Another possible approach to tackle effector function could arise from the great number of mutant lines of plants, mainly *Arabidopsis*, which have a specific phenotype associated with a determined cellular process. Plants over-expressing pathogen effectors could be phenotypically screened against known *Arabidopsis* mutants allowing the association of an effector with a cellular process (Figure 2B).

A study in Phytoplasma successfully used such an approach. Phytoplasma are insect-transmitted bacterial pathogens that cause severe phenotypic changes in the host morphology to enhance the fitness of their insect vectors. These phenotypic changes include aberrant flower development and abnormal host tissue growth and were proposed to be caused by the action of effector proteins secreted by phytoplasma. Thus, 56 candidate effectors of Aster Yellows phytoplasma strain ‘Witches Broom’ (AY-WB) were expressed in *Arabidopsis* and screened for aberrant flowering development. This approach allowed the identification of one effector secreted AY-WB protein 54 (SAP54), that indeed causes aberrant flowering development when over-expressed in *Arabidopsis* [73, 74]. Another effector, SAP11, was identified in this screen by causing abnormal growth development in *Arabidopsis*. SAP11 was then shown to target the host nucleus and to enhance colonization of the AY-WB insect vector *Macrosteles quadrilineatus* when over-expressed in *Arabidopsis* [75]. The phenotype of *Arabidopsis* expressing SAP11 had similarities with plants over-expressing miR319, a microRNA induced by phosphate starvation responses. Identifying two over-expressing lines, which show altered phosphate starvation responses, suggests similar cellular processes are being targeted [76]. Thus, we propose that systematic phenotypic screens of *Arabidopsis* lines, over-expressing pathogen effectors, could yield important leads to infer effector function.

In an approach that is akin to phenocopy assays, mutants with altered specific cellular process could be complemented with pathogen effectors and screened phenotypically. Complementation of mutant phenotypes would allow identification of direct links between effectors and the cellular mechanism they target.

### Functional proteomics and next-generation sequencing

One of the first steps for the exploration of effector function is the identification of its target(s). A large number of studies have been focused on individual effectors. However, to fully understand plant–pathogen interactions, large sets of identified effectors need to be screened. In the *A. thaliana*–*H. arabidopsidis/P. syringae* pathosystem, a large-scale Yeast-two Hybrid (Y2H) screen of 83 effectors from both pathogens suggested that effectors are likely to target a set of interconnected plant proteins, which are conserved between plant pathosystems [51]. The limitation with such screens, however, is a lack of mechanistic information describing effector activity *in vivo*. We surmise that the deployment of -omics technologies to study effector activity in plants forms a complementary, but as of yet untested approach to study effector functions. Given the availability and decreasing cost of NGS technology, effectors and effector-triggered modifications can be dissected one by one to unveil the mechanisms of effector triggered susceptibility (ETS). Effectors can be expressed *in planta*, after which functional analysis could be pursued by means of proteomics (after enrichment for specific PTMs, or organelles) and NGS-based functional genomics (RNA-seq, CHIP-seq, Methyl-seq) (Figure 2C). In *Arabidopsis*, RNAseq analysis of transgenic plants overexpressing bacterial effector SAP11 showed the suppression of a set of host genes involved in plant immune responses [76]. Transcriptomics-based analyses of tomato–*P. syringae* interactions, combined with reporter gene assays, revealed a number of host genes specific to ETI, including a novel kinase [77]. If implemented successfully, specific effector-induced changes and nuclear processes should be identifiable and investigated further. These, in turn, would reveal effector functions in a comprehensive way and improve our understanding of plant–pathogen interactions to greater detail.

## Concluding remarks

We continuously acquire knowledge about the plant immune system and plant–pathogen interactions, but there is still a lot unknown. The interconnected nature of the plant immune system highlights the necessity to expand the toolset with which to interrogate plant immune signalling. A recent model suggests that activation of R genes results in the induction of reactive oxygen species (ROS) production and the modification of Red/Ox balance, causing growth inhibition [78]. Moreover, in *Arabidopsis*, several genes required for the progression of cell cycle were reported to affect the expression of plant R genes and contribute to ETI [79, 80]. The cytoskeleton was recently identified as being linked to PTI and involved in stomatal closure [81], PAMP receptors endocytosis and MAPK signalling [82]. It was also shown to be disrupted by pathogen effectors [83–85]. Finally, the nucleolus appears to be targeted by various plant pathogens including viruses ([86–88] and is therefore of great interest. Targeted studies of nucleolar effectors as described above may help identify nucleolar processes that affect plant immunity [89–93].

Here, we have described a variety of well-known nuclear processes involved in plant immune responses. Because only few pathogen effectors that target these processes are known, we have suggested several approaches for the large-scale identification of effector function involved in nuclear immunity processes. It should be noted, however, that there are many other cellular processes/components that are thought to be involved in plant immunity. The increasing number of examples implies not only the complexity of plant immune system but also its pluri-sided and interconnected nature. For better understanding of plant–pathogen interactions, it is important to generate an authentic network view of plant immune signalling. This will allow us to dissect the critical processes, model them [3] and, subsequently, manipulate plant immunity.

**Key points**

- The plant immune system is regulated by a vast signalling network, much of which is located in the host nucleus. The immune signalling network intersects with other key developmental and regulatory processes within the cell.
- Pathogens suppress host immunity by secreting arrays of proteins (effectors) that perturb host signalling and other cellular events.
- Next-generation sequencing and functional genomics approaches have helped (i) identify nuclear processes that underpin immunity and (ii) identify and group pathogen effector classes, some of which target processes that reside in the host nucleus.
- The availability of host and pathogen genome sequences, systems level information on immunity-associated processes and increasingly accessible next-generation -omics platforms, will inspire new functional assays to dissect the host immune system.
